# Protection of Iron-Induced Oxidative Damage in Neuroblastoma (SH-SY5Y) Cells by Combination of 1-(N-Acetyl-6-aminohexyl)-3-hydroxy-2-methylpyridin-4-one and Green Tea Extract

**DOI:** 10.1155/2021/5539666

**Published:** 2021-04-20

**Authors:** Nittaya Chansiw, Kanokwan Kulprachakarn, Narisara Paradee, Adchara Prommaban, Somdet Srichairatanakool

**Affiliations:** ^1^School of Medicine, Mae Fah Luang University, Chiang Rai 57100, Thailand; ^2^Research Institute for Health Science, Chiang Mai University, Chiang Mai 50200, Thailand; ^3^Oxidative Stress Cluster, Department of Biochemistry, Faculty of Medicine, Chiang Mai University, Chiang Mai 50200, Thailand

## Abstract

Iron is a crucial trace element and essential for many cellular processes; however, excessive iron accumulation can induce oxidative stress and cell damage. Neurodegenerative disorders, such as Alzheimer's disease and Parkinson's disease, have been associated with altered iron homoeostasis causing altered iron distribution and accumulation in brain tissue. This study aims to investigate the protective effect of 1-(*N-*acetyl-6-aminohexyl)-3-hydroxy-2-methylpyridin-4-one (CM1) in combination with green tea extract (GTE) on iron-induced oxidative stress in neuroblastoma (SH-SY5Y) cells. Cells were cultured in medium with or without ferric chloride loading. Their viability and mitochondrial activity were assessed using MTT and JC-1 staining methods. Levels of the cellular labile iron pool (LIP), reactive oxygen species (ROS), and lipid-peroxidation products were determined using calcein acetoxymethyl ester, 2′,7′-dichlorohydrofluorescein diacetate, and TBARS-based assays, respectively. The viability of iron-loaded cells was found to be significantly increased after treatment with CM1 (10 *µ*M) for 24 h. CM1 co-treatment with GTE resulted in a greater protective effect than their monotherapy. Combination of CM1 and GTE also reduced mitochondrial disruption and LIP content and ROS and TBARS production. In conclusion, the combination of CM1 and GTE exhibits protection against iron-induced oxidative stress in neuroblastoma cells.

## 1. Introduction

Iron is an essential element which is involved in many cellular processes including oxygen transport, oxygen sensing, electron transduction, energy metabolism, and DNA synthesis. However, it also participates in the undesirable Fenton reaction by reacting with hydrogen peroxide to generate the hydroxyl radical and other reactive oxygen species (ROS) [1, 2]. ROS attacks polyunsaturated fatty acids, causing peroxidation, which in turn causes disorganization and dysfunction [3, 4]. Lipid peroxidation is used as an indicator of oxidative stress in cells and tissues and can be monitored by the formation of byproducts such as malondialdehyde (MDA). Normally, iron is efficiently taken up by cells using the divalent metal transporter 1, this process being tightly controlled. However, an abnormally high iron uptake will increase the cellular labile iron pool (LIP), which could then lead to oxidative damage and cell death [1, 5]. During the natural process of aging, different iron complexes accumulate in brain regions that cause motor and cognitive impairment [4, 6, 7]. Neurodegenerative diseases, such as Alzheimer's disease and Parkinson's disease, are associated with a change in iron homoeostasis resulting in altered cellular iron distribution and accumulation [8–10].

Interestingly, iron chelators, such as 7,8-dihydroxy-4-((methylamino)methyl)-2H-chromen-2-one,dihydroxamate peptide and deferiprone (DFP), are effective in removing excess intracellular iron pools, thereby inhibiting lipid peroxidation and maintaining the normal mitochondrial membrane potential of neuronal cells [11–13]. Such chelators have potential to provide neuroprotection in neurodegenerating disease [12, 13]. Tea extracts have been separated to attenuate 6-hydroxydopamine-induced neuroblastoma (SH-SY5Y) cell death via the anti-oxidant and iron-chelating properties of polyphenols, particularly epigallocatechin-3-gallate (EGCG) [14, 15].

1-(*N*-Acetyl-6-aminohexyl)-3-hydroxypyridin-4-one (CM1) is an orally active bidentate iron chelator firstly synthesized by Pangjit et al. [16]. It is a DFP analogue but is characterized by a higher level of lipid solubility [17]. In a previous study, CM1 was demonstrated to effectively bind iron under biological conditions. It also reduces plasma non-transferrin bound iron and labile plasma iron [18]. Importantly, the compound shows low toxicity [19, 20]. However, there are published reports of this compound processing a neuroprotective effect. Green tea extract (GTE) prepared from tea (*Camellia sinensis*) leaves is predominantly comprised of polyphenolic catechins, of which EGCG is the most abundant [21, 22]. Interestingly, EGCG has been reported to protect AS-PC12 and SH-SY5Y cells against iron-induced oxidative stress [23, 24]. Likewise, we have previously demonstrated that GTE exhibits anti-oxidant, free radical-scavenging, and iron chelating activities in iron-loaded thalassemia mice [25]. In the present study, we reported the protective effect of CM1 in combination with GTE against iron-induced oxidative stress in SH-SY5Y cells.

## 2. Materials and Methods

### 2.1. Chemicals and Reagents

Calcein acetoxymethyl (CA-AM) and JC-1 dye were purchased from Invitrogen^®^ Corporation (Carlsbad, CA, USA). Furthermore, 2′,7′-dichlorodihydrofluorescein diacetate (DCFH-DA), dimethyl sulfoxide (DMSO), epigallocatechin-3-gallate (EGCG), ferric chloride (FeCl_3_), 1,1,3,3-tetramethoxypropane (TMP), thiobarbituric acid (TBA), *α*-tocopherol (TC), thiobarbituric acid (TBA), and 3-(4,5-dimethyl-2-thiazolyl)-2,5-diphenyl-2-*H*-tetrazolium bromide (MTT) were purchased from Sigma-Aldrich Chemicals Company (St. Louis, MO, USA). Bradford's reagent was purchased from Bio-Rad Laboratories, Inc. (Hercules, CA, USA). Dulbecco's Modified Eagle Medium (DMEM), Ham's F12 Nutrient Mixture, fetal bovine serum (FBS), phosphate-buffered saline (PBS) pH 7.0, and penicillin-streptomycin were purchased from Gibco Laboratories (Gaithersburg, MD, USA).

### 2.2. Substances

CM1 was synthesized according to literature procedure [17]. GTE was locally prepared from fresh tea (*Camellia sinensis*) leaves using a microwave-based method to dry the leaves and for the inactivation of polyphenol oxidase. Subsequently, EGCG was found to be 24% (*w*/*w*) of the final product [16]. GTE was freshly dissolved in 1% (v/v) DMSO each day and concentrations were expressed as mg/mL and EGCG equivalents. Among iron sources, FeCl_3_ and FeCl_2_ were used as a redox iron for loading into cells to induce iron overload [26–28]. In this study, the iron solution (100 mM) was initially prepared by dissolving 16.22 mg of FeCl_3_ in 1.0 mL of 500 mM citric acid and diluted in working medium to achieve the final concentration of 2 mM.

### 2.3. Cell Culture

Human neuroblastoma (SH-SY5Y) cell line was purchased from the American Type Culture Collection (ATCC® CRL2266™, Manassas, VA, USA). Cells were cultured in DMEM and Ham's F12 Nutrient Mixture (a ratio of 1 : 1, v/v) supplemented with 20% (v/v) FBS, 100 U/mL penicillin, and 100 *µ*g/mL streptomycin. Cells were then maintained at 37°C in a 5% CO_2_ incubator.

### 2.4. Cell Toxicity Study

The toxic effects of CM1 and GTE were investigated by measuring the viability of SH-SY5Y cells using the MTT assay [29]. Briefly, SH-SY5Y cells (2 × 10^4^/well) were seeded onto 96-well plates and left for 24 h. The cells were then treated with 1% DMSO (control), CM1 (10–100 *μ*M), and GTE (10–100 mg/mL equivalent to 5.24–52.4 *μ*M EGCG, respectively) for 24 or 48 h. Then, a MTT solution (5 mg/mL) was added to the cells and they were incubated for a further 4 h at 37°C. The medium was removed and 1% DMSO solution was added to the well plates in order to dissolve the formazan crystals. The optical density (OD) was measured at a wavelength of 540 nm/630 nm using a 96-well microtiter plate reader (BioTek Synergy H4 Hybrid Reader, BioTek Instruments Inc., VT, USA). Untreated cells were used as a control group.

Under iron-overload conditions, SH-SY5Y cells (2 × 10^4^/well) were seeded onto 96-well plates for 24 h. They were incubated in medium containing 2 mM FeCl_3_ for 24 h and then washed three times with PBS pH 7.0. The cells were then treated with CM1 and GTE at the indicated concentrations and 1% DMSO (control) for 24 h. Finally, cell viability of the treated cells was estimated using the MTT assay as described above.

### 2.5. Measurement of Mitochondrial Membrane Potential

Disruption of mitochondrial membrane potential (MMP, ΔΨ_m_) is one of the intracellular events leading to apoptosis. The 5,5ʹ,6,6ʹ-tetrachloro-1,1ʹ,3,3ʹ-tetraethylbenzimidazolyl carbo-cyanine iodide (JC-1) dye, which is a lipophilic cationic carbocyanine fluorescent probe used to identify changes in the MMP, selectively enters into the mitochondria and reversibly changes color from green to red as the MMP changes. In healthy cells with a high MMP level, JC-1 spontaneously forms complexes with an intense red fluorescence. In contrast, with apoptotic or unhealthy cells with low MMP, JC-1 remains in the monomeric form and displays only green fluorescence. SH-SY5Y cells (5 × 10^4^/well) were seeded onto a 24-well plate for 24 h, the medium was removed, and the cells were loaded with the 2 mM FeCl_3_. After 24 h, the medium was removed and the cells were washed two times with PBS. The cells were then treated with 1% DMSO (control), CM1 (10, 20, and 40 *µ*M), GTE (10, 20, and 40 mg/mL equivalent to 5.24, 10.48, and 20.96 *μ*M EGCG, respectively), and combinations of CM1 and GTE for 24 h. After discarding the medium, the treated cells were washed two times with PBS, and JC-1 dye was added and incubated in the dark at 37°C for 20 min. After this short incubation, fluorescence intensity (FI) at an excitation wavelength of 510 nm and an emission wavelength of 527 nm was recorded by flow cytometry (DxFLEX Flow Cytometer, Beckman Coulter Life Sciences, IN, USA) [30]. The resulting FI was a measure of the percentage change of MMP.

### 2.6. Measurement of Labile Iron Pool

The level of the intracellular labile iron pool (LIP) was investigated using the fluorescence calcein quenching technique [31]. In principle, CA-AM passes through the cell membrane and reacts with cytosolic unspecific esterases to produce calcein, a hydrophilic fluorochrome which is retained within the cytosol of the cells. Calcein fluorescence is quenched in the presence of labile iron, the degree of quenching providing an estimate of the amount of chelatable iron present in the cytosol. Briefly, SH-SY5Y cells (1 × 10^4^/well) were seeded onto a black 96-well plate for 24 h, and the medium was replaced with the medium containing 2 mM FeCl_3_. After incubation for 24 h, the medium was removed and the cells were washed three times with PBS. The cells were then treated with 1% DMSO, different concentrations of CM1 (10, 20, and 40 *μ*M), EGCG (10, 20, and 40 *μ*M), GTE (10, 20, and 40 mg/mL equivalent to 5.24, 10.48, and 20.96 *μ*M EGCG, respectively), and combinations of the EGCG and GTE with 10 *μ*M CM1 for 24 h. After discarding the medium, the treated cells were washed with PBS two times, and CA-AM (1 *µ*M in PBS) was added and incubated for 30 min at 37°C. After calcein loading, the cells were washed three times with PBS and FI measured at an excitation wavelength of 488 nm and an emission wavelength of 515 nm. The cells without iron loading were used as a control group (100% FI) to calculate the percentage change of FI after the various treatments.

### 2.7. Analysis of Reactive Oxygen Species

ROS production was investigated using DCFH-DA staining and then analyzed by spectrofluorometric analysis [32]. DCFH-DA diffuses into the cells and is hydrolyzed by cellular esterase to produce 2′,7′-dichlorofluorescein (reduced form), which will then be subsequently oxidized by existing ROS to a green-fluorescence 2′,7′-dichlorofluorescein (DCF) product. Notably, high green FI indicates a high level of intracellular ROS. SH-SY5Y cells (2 × 10^4^/well) were seeded onto a 96-well plate for 24 h, then the medium was removed, and the cells were loaded with the medium containing 2 mM FeCl_3_ (24 h). The cells were then incubated with 1% DMSO (control), *α*-tocopherol (0.46 *μ*M), different concentrations of CM1 and GTE, and combinations of the two for 24 h at 37°C. The cells were then treated with DCFH-DA solution (10 *µ*M) at 37°C for 30 min. The cells were washed twice with PBS solution and FI was measured at an excitation wavelength of 485 nm and an emission wavelength of 530 nm using a 96-well plate spectrofluorometer (BioTek Synergy H4 Hybrid Reader, BioTek Instruments Inc., VT, USA) for 1 h to establish kinetic measurement. The FI value is directly proportional to the amount of ROS present in the cells. The iron-loaded cells treated with 1% DMSO were used as a control group (100% FI) to calculate the percentage change of FI after the various treatments.

### 2.8. Quantification of Lipid-Peroxidation Products

TBA reacts with aldehydes that are released from the lipid peroxidation of membrane phospholipids to yield a pink-colored product which can be determined photometrically. SH-SY5Y cells (1.5 × 10^6^/well) were seeded onto a 6-well plate for 24 h and then iron-loaded for further 24 h. The cells were then incubated with 1% DMSO (control), *α*-tocopherol (0.46 *μ*M), different concentrations of CM1 and GTE, and combinations of the two for 24 h at 37°C. The treated cells were harvested, washed with PBS twice, and centrifuged at 300 g for 10 min. The cell pellets were collected and resuspended in PBS. Cell lysates were prepared by placing cells in a Bioruptor water bath sonicator (the condition set at 24 sec ON, 24 sec OFF) housed in a cold room (5–6°C) for 2–3 min and centrifuged at 10,000 g for 15 min. Total protein content was determined using Bradford's reagent. The lipid-peroxidation products were measured colorimetrically using the TBARS assay. Cell lysate or standard MDA was mixed with 1% meta-phosphoric acid and 0.67% TBA and incubated in a water bath at 95°C for 60 min. After being cooled, *n*-butanol was added and the mixtures were centrifuged at 4500 rpm for 5 min. The OD of the *n*-butanol phase was measured at 535 nm using a double beam UV-VIS spectrophotometer. Standard curves were prepared over the concentration range 0.312–20 *µ*M TMP.

### 2.9. Statistical Analysis

The results were expressed as mean ± standard error of the mean (SEM) of three independent measurements. Statistical analysis was determined using one-way analysis of variance (ANOVA), after which a post-hoc test was applied. Values of *P* < 0.05 were considered to be significantly different.

## 3. Results

### 3.1. Toxicity on SH-SY5Y Cells

**Figure 1:**
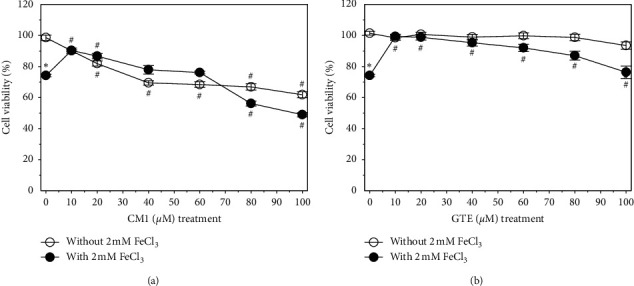
Viability of SH-SY5Y cells after being exposed to treatments with or without 2 mM FeCl_3_. Cells were then treated with CM1 (10–100 *μ*M) (a) and GTE (10, 20, 40, 60, 80, and 100 mg/mL equivalent to 5.24, 10.5, 20.48, 31.4, 41.9, and 52.4 *μ*M EGCG, respectively) (b) for 24 h. Data obtained from three independent duplicate experiments are expressed as mean ± SEM values. ^*∗*^*P* < 0.05 when compared to the untreated cells without iron loading; ^#^*P* < 0.05 when compared to the cells without treatment.

Cell viability was significantly increased in a concentration-dependent manner when SH-SY5Y cells were treated with CM1 for 24 h, in which 10 and 20 *μ*M CM1 treatments improved the viability of iron-loaded cells, while 80 and 100 *μ*M CM1 were found to be toxic to the cells and decreased the degree of viability significantly (Figure 1(a)). In comparison, all GTE concentrations were found to be non-toxic to normal neuroblastoma cells (cell viability >90%) (Figure 1(b)). Similarly, GTE in the range of 10–80 mg/mL (5.2–41.9 *μ*M EGCG equivalent) improved viability of the iron-loaded cells. However, the degree of cell viability was decreased in a concentration-dependent manner. CM1 at 10 *µ*M and GTE in the range of 10–40 mg/mL (5.2–21 *µ*M EGCG equivalents) were selected for subsequent experiments.

### 3.2. Effect on Mitochondrial Function

**Figure 2:**
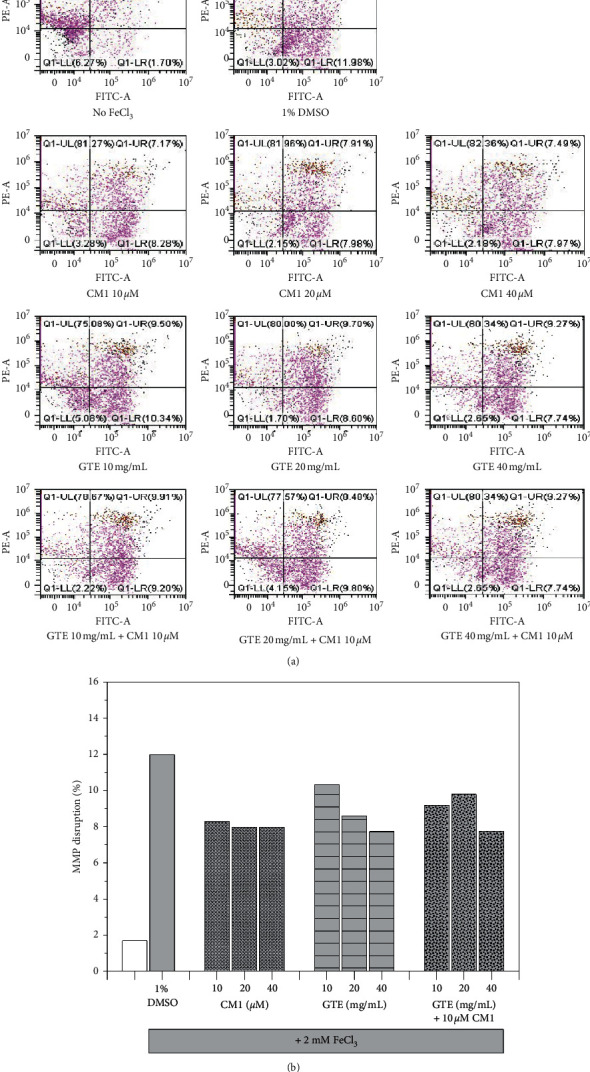
Mitochondrial membrane disruption in SH-SY5Y cells after being exposed to treatments with or without 2 mM FeCl_3_. Cells were then treated with 1% DMSO, CM1 (10–40 *μ*M), GTE (10, 20, and 40 mg/mL equivalent to 5.24, 10.48, and 20.96 *μ*M EGCG, respectively) and a combination of CM1 and GTE for 24 h. Data are expressed as dot-plot graphs (a) and histogram of triplicate experiments (b).

The iron loading of SH-SY5Y cells was found to disrupt mitochondrial function (Figure 2). Interestingly, GTE (10–40 mg/mL) monotherapy lowered the percentage of MMP disruption in a dose-dependent manner, whereas CM1 monotherapy also induced a change in MMP, but not in a dose-dependent manner. Co-treatment of CM1 and GTE did not promote an enhanced MMP disruption when compared to monotherapy. The results suggest that both CM1 and GTE are able to chelate redox active iron in the mitochondrial compartment and exert a protective effect on oxidative neuroblastoma cell damage.

### 3.3. Effect on Labile Iron Pool

**Figure 3:**
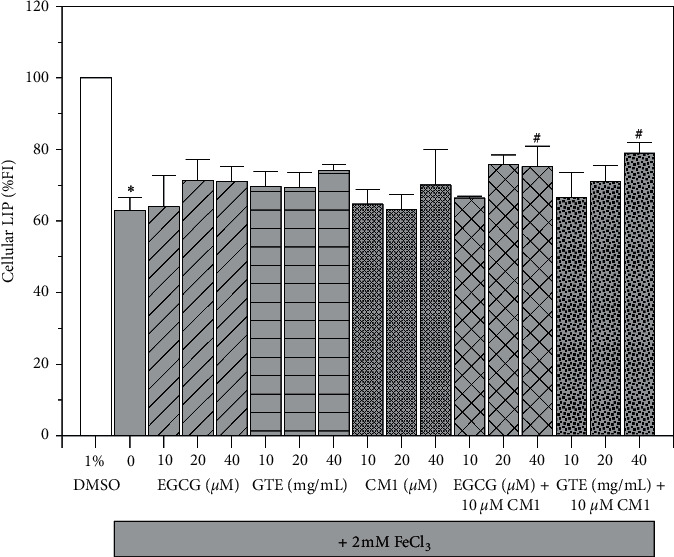
Levels of LIP in SH-SY5Y cells with iron loading condition. SH-SY5Y cells were being exposed with or without 2 mM FeCl_3_. After that, the cells were then treated with 1% DMSO, CM1 (10–40 *μ*M), EGCG (10–40 *μ*M), GTE (10, 20, and 40 mg/mL equivalent to 5.24, 10.48, and 20.96 *μ*M EGCG, respectively), and combinations for 24 h. Data obtained from three independent duplicate experiments are expressed as mean ± SEM values. ^*∗*^*P* < 0.05 when compared to the untreated cells without iron loading; ^#^*P* < 0.05 when compared to the iron-loaded cells treated with 1% DMSO.

The amount of intracellular LIP is inversely proportional to fluorescence signal assayed using the calcein fluorescence quenching method. The loading of 2 mM FeCl_3_ resulted in significant iron accumulation in SH-SY5Y cells when compared to healthy cells monotherapy of CM1 (10–20 *µ*M) and GTE did not lead to LIP reduction in iron-loaded cells while higher concentrations slightly decreased the LIP levels (*P* > 0.05). Moreover, combined EGCG treatment with 10 *μ*M CM1 slightly enhanced the chelation of LIP, while combined GTE treatment with the two chelators tended to increase the degree of chelation when compared to their monotherapies (Figure 3). It is possible that EGCG and other active phytochemicals in GTE, together with CM1, could cooperatively remove LIP in neuroblastoma cells with iron overload.

### 3.4. Effect on ROS Production

**Figure 4:**
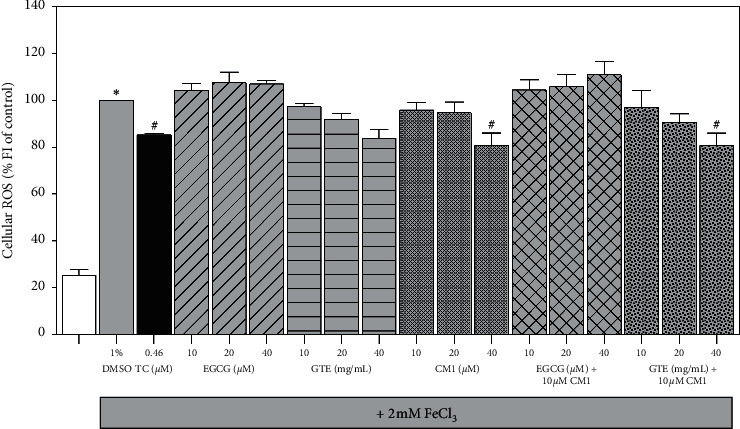
Levels of ROS in SH-SY5Y cells after being exposed to treatments with or without 2 mM FeCl_3_. Cells were then treated with 1% DMSO, TC (0.46 *μ*M), CM1 (10–40 *μ*M), EGCG (10–40 *μ*M), GTE (10, 20, and 40 mg/mL equivalent to 5.24, 10.48, and 20.96 *μ*M EGCG, respectively), and combinations for 24 h. Data obtained from three independent duplicate experiments are expressed as mean ± SEM values. ^*∗*^*P* < 0.05 when compared to the untreated cells without iron loading; ^#^*P* < 0.05 when compared to the iron-loaded cells treated with 1% DMSO.

Obviously, the percentage of ROS production was significantly increased in iron-loaded SH-SY5Y cells when compared to healthy cells; however, the level was significantly decreased by *α*-tocopherol (0.46 *μ*M) and then used as a reference fat-soluble anti-oxidant (Figure 4). Monotherapy involving EGCG (10–40 *μ*M) showed ineffective free-radical scavenging activity; in contrast, CM1 (10–40 *μ*M) and GTE (10–40 mg/mL) displayed effective free-radical scavenging activity in a concentration-dependent manner, in which the 40 *μ*M CM1 was found significantly different. In combined therapy, only 40 mg/mL GTE along with 10 *µ*M CM1 resulted in a significant decrease in ROS production while other combinations did not. The results imply that EGCG and other active phytochemicals in GTE together with CM1 could cooperate to scavenge ROS in neuroblastoma cells with iron overload.

### 3.5. Effect on Lipid-Peroxidation Reaction

**Figure 5:**
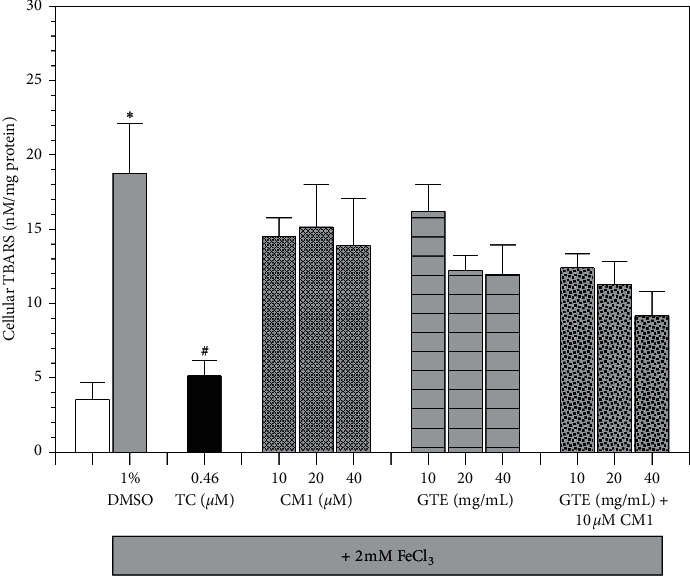
Levels of TBARS in SH-SY5Y cells after being exposed to treatments with or without 2 mM FeCl_3_. Cells were treated with 1% DMSO, TC (0.46 *μ*M), CM1 (10–40 *μ*M), GTE (10, 20, and 40 mg/mL equivalent to 5.24, 10.48, and 20.96 *μ*M EGCG, respectively), and a combination of GTE and CM1 for 24 h. Data obtained from three independent duplicate experiments are expressed as mean ± SEM values. ^*∗*^*P* < 0.05 when compared to the untreated cells without iron loading; ^#^*P* < 0.05 when compared to the iron-loaded cells treated with 1% DMSO.

Lipid peroxidation, which mostly occurs on membrane phospholipids, is routinely catalyzed by ROS to generate byproducts including 4-hydroxynonenal, isoprostane, and aldehydes (such as malondialdehyde) that can react with TBA and yield pink-colored TBARS. Apparently, FeCl_3_ loading catalyzed ROS generation and significantly increased TBARS levels in SH-SY5Y cells, while *α*-tocopherol treatment (0.46 *μ*M) was effective in significantly lowering the increase of TBARS levels (Figure 5). Treatments with CM1 and GTE also reduced the cellular TBARS levels in which the GTE was more effective. Importantly, a combination treatment of GTE with 10 *μ*M CM1 was more effective than GTE monotherapy in decreasing the TBARS levels in a concentration-dependent manner. The results indicated that antioxidants, such as *α*-tocopherol and green tea extract, directly scavenged ROS and subsequently prevented lipid-peroxidation reactions in neuroblastoma cells. Thus, they were found to be more effective in this manner than other iron chelators such as CM1.

## 4. Discussion

Iron is a crucial trace element required for many important brain functions including energy metabolism, synaptic plasticity, myelination, and synthesis of neurotransmitters [33, 34]. Many neurodegenerative diseases have been associated with the disruption of brain iron homeostasis resulting in an accumulation of brain iron and the provoking of chronic neuroinflammation [4, 35, 36]. Importantly, a redox-active iron can generate ROS via a Fenton reaction, which is one of the mechanisms believed to play an important role in the pathogenesis of Alzheimer's disease and Parkinson's disease [37–40]. Indeed, the levels of generated ROS are significantly higher in the brains of Alzheimer's patients when compared to healthy control brains [41, 42]. Furthermore, many studies have reported that oxidative stress promotes neurotoxic oligomerization of A *β* peptides and tau tangles [43, 44]. ROS also activated microglia to release pro-inflammatory cytokines that are known to cause chronic inflammation [45, 46]. On the other hand, patients with Parkinson's disease have been observed with high total iron concentrations in the substantia nigra that might be associated with increased divalent metal iron transporter 1 activity or decreased ferroxidase activity of ceruloplasmin, potentially both of which would increase total intracellular iron as has been reported in patients and animal models [38, 47, 48]. The mechanism of iron-induced neuronal cell death has been reported by Bautista and colleagues [49]. They found increases of extracellular signal-regulated kinase (ERK) and protein kinase B (PKB also known as Akt) activities and a reduction of dual specificity tyrosine-phosphorylation-regulated kinase 1B (DyrK1B) after FeCl_3_ treatment. Furthermore, they also recorded activated transcription factors, such as c-Jun and phosphor-Smad1/5, suggesting that the presence of a high concentration of iron could increase the vulnerability of neurons to oxidative stress.

Since high iron accumulation is a cause of neuronal cell death, iron chelation might be considered a useful form of therapy and has therefore received a significant amount of attention. Previous studies have reported on the neuroprotective effect of iron chelators such as DFP, deferoxamine, and others [50–53]. However, these chemical drugs may exhibit adverse effects suggesting that non-toxic products are required. Our lead compound, CM1, is a synthetic bidentate chelator that is more lipophilic than DFP and can bind the iron efficiently [17]. Interestingly, the chelator is able to chelate NTBI in thalassemic serum, but is not toxic to peripheral blood mononuclear cells, hepatocytes, and myocytes *in vitro* [20]. Due to the high cost of chemical drugs, we are increasingly interested in alternative therapies that employ natural products. Several studies have demonstrated that green tea has neuroprotective functions, as well as non-toxic and positive synergistic effects. Green tea contains high amounts of polyphenolic catechins including EGCG, epigallocatechin, epicatechin, and epicatechin-3-gallate [54, 55]. Many studies have shown that EGCG exhibits a range of anti-oxidant, anti-inflammatory, anti-microbial, anti-cancer, and neuroprotective properties [56–58]. Interestingly, EGCG can cross the blood brain barrier and display its neuroprotective effect [21, 59]. In order to investigate the neuroprotective effect of an oral iron chelator, CM1 in combination with GTE under iron-overloaded conditions in neuroblastoma cells, such as SH-SY5Y cells, was prepared and its protective effect was assessed against iron-induced oxidative stress. The results demonstrate that high concentrations of CM1 were toxic to the cells because iron depletion can affect many important processes of the cells. In combination treatments, we combined a non-toxic dose of CM1 with GTE at various concentrations. It was found that co-treatment of CM1 and GTE exhibited greater activities by saving cell viability and could decrease the levels of labile iron pool, ROS, and lipid-peroxidation products presented as TBARS. The findings suggest that CM1 and green tea extract exhibit a synergistic action to protect the neuroblastoma cells from iron-induced oxidative stress by chelating excessive iron and reducing ROS via anti-oxidant properties. However, studies involving both animals and humans require further elucidation for applications in alternative medicine.

## 5. Conclusion

A combination of CM1 and green tea extract exhibited enhanced neuroprotective action against iron-induced oxidative stress in neuroblastoma cells by improving cell viability and mitochondrial function, and reducing levels of labile iron pool, reactive oxygen species, and lipid-peroxidation products.

## Data Availability

The data used to support the findings of this study are available within the article and from the corresponding author upon request.

## Abbreviations

ANOVA: One-way analysis of variance
CA-AM: Calcein acetoxymethyl
CM1: 1-(*N-*acetyl-6-aminohexyl)-3-hydroxy-2-methylpyridin-4-one
DCFH-DA: 2′,7′-Dichlorodihydrofluorescein diacetate
DFP: Deferiprone
DMEM: Dulbecco's Modified Eagle Medium
DMSO: Dimethyl sulfoxide
DyrK1B: Dual specificity tyrosine-phosphorylation-regulated kinase 1B
EGCG: Epigallocatechin-3-gallate
ERK: Extracellular signal-regulated kinase
FBS: Fetal bovine serum
FI: Fluorescence intensity
GTE: Green tea extract
LIP: Labile iron pool
MDA: Malondialdehyde
MTT: 3-(4,5-Dimethyl-2-thiazolyl)-2,5-diphenyl-2-*H*-tetrazolium bromide
MMP: Mitochondrial membrane potential
OD: Optical density
PBS: Phosphate-buffered saline
PKB: Protein kinase B
ROS: Reactive oxygen species
SEM: Standard error of mean
TBA: Thiobarbituric acid
TBARS: Thiobarbituric acid-reactive substances
TMP: 1,1,3,3-Tetramethoxypropane
UV-VIS: Ultraviolet-visible.
